# The Prevalence of Frailty and its Associated Factors in Japanese Hemodialysis Patients

**DOI:** 10.14336/AD.2017.0429

**Published:** 2018-04-01

**Authors:** Hidemi Takeuchi, Haruhito A. Uchida, Yuki Kakio, Yuka Okuyama, Michihiro Okuyama, Ryoko Umebayashi, Kentaro Wada, Hitoshi Sugiyama, Ken Sugimoto, Hiromi Rakugi, Jun Wada

**Affiliations:** ^1^Department of Nephrology, Rheumatology, Endocrinology and Metabolism, Okayama University Graduate School of Medicine, Dentistry and Pharmaceutical Sciences, Okayama, Japan.; ^2^Department of Chronic Kidney Disease and Cardiovascular Disease, Okayama University Graduate School of Medicine, Dentistry and Pharmaceutical Sciences, Okayama, Japan.; ^3^Department of Cardiovascular Surgery, Okayama University Hospital, Okayama, Japan.; ^4^Division of Nephrology and Dialysis, Department of Internal Medicine, Nippon Kokan Fukuyama Hospital, Hiroshima, Japan.; ^5^Department of Human Resource Development of Dialysis Therapy for Kidney Disease, Okayama University Graduate School of Medicine, Dentistry and Pharmaceutical Sciences, Okayama, Japan.; ^6^Department of Geriatric and General Medicine, Osaka University Graduate School of Medicine, Osaka, Japan.; ^7^Department of Internal Medicine, Innoshima General Hospital, Hiroshima, Japan

**Keywords:** Frailty, Dialysis, Frailty phenotype

## Abstract

The population undergoing dialysis is aging worldwide, particularly in Japan. The clinical condition of frailty is the most problematic expression in the elderly population. Potential pathophysiological factors of frailty present in patients with CKD and are accentuated in patients with ESRD. The aim of this study was to identify the prevalence and predictors of frailty in Japanese HD patients. This study was a multicenter, cross-sectional and observational investigation conducted at 6 institutions. To evaluate frailty, the modified Fried’s frailty phenotype adjusted for Japanese as the self-reported questionnaire was used. Of the 542 patients visiting each institution, 388 were enrolled in this study. In total, 26.0% of participants were categorized as not-frailty, 52.6% as pre-frailty and 21.4% as frailty. The prevalence of frailty increased steadily with age and was more prevalent in females than in males and the subjects with frailty received polypharmacy. A multivariate logistic regression analysis revealed that the factors independently associated with frailty were the following: female gender (odds ratio [OR] = 3.661, 95% confidence interval [CI] 1.398-9.588), age (OR = 1.065, 95% CI 1.014-1.119), age ≥ 75 years old (OR = 4.892, 95% CI 1.715-13.955), body mass index (BMI) < 18.5 (OR = 0.110, 95% CI 0.0293-0.416), number of medications being taken (OR = 1.351, 95% CI 1.163-1.570), diabetes mellitus (DM) (OR = 2.765, 95% CI 1.081-7.071) and MNA-SF ≤ 11 (OR = 7.405, 95% CI 2.732-20.072). Frailty was associated with the accumulation of risk factors. The prevalence of frailty in Japanese patients with HD was relatively lower than that previously reported in Western developed countries; however, it was extremely high compared to the general population regardless of age. Our findings suggest that frailty might be associated with an increase in the prevalence of adverse health outcomes in patients with HD.

Recently, the lifespan of the global population has begun rapidly increasing. Globally, life expectancy at birth increased from 61.7 years in 1980 to 71.8 years in 2015 [1]. However, by contrast, the healthy life expectancy at birth was 60.9 years for men and 64.9 years for women in 2015. The gap between life expectancy and healthy life expectancy represents years of functional health lost [2]. This gap has thus become a major concern worldwide.

A particularly problematic issue plaguing the elderly population is the clinical condition of frailty. Frailty is considered to indicate the end of healthy life expectancy and develops as a consequence of the age-related decline in physiological systems, resulting in increasing individual vulnerability to health status changes [3]. Fried et al. developed a frailty phenotype as a convenient definition of frailty [4]. Frailty leads to social endpoints, such as hospitalization, fall and worsening activity of daily life (ADL), as well as hard endpoints, such as mortality. This report brought frailty global focus. The factors associated with presence of frailty were aging, female gender, race, socioeconomic state [3], smoking, obesity, shrinking, a history of cardiovascular disease (CVD), bone fracture, falling, chronic obstructive pulmonary disease, diabetes mellitus (DM), depression [5], undernutrition [6], dementia [7], malignancy [8], chronic kidney disease (CKD), and end-stage renal disease (ESRD) [9].

The ESRD population is also aging worldwide, particularly in Japan. The mean age of the total Japanese ESRD population is 67.2 years, and 61.8% were ≥ 65 years old, and 30.3% were ≥ 75 years old at the end of 2013 [10]. Potential pathophysiological factors of frailty present in patients with CKD, and even more are found in patients with ESRD [11]. According to Fried’s definition, the frailty status has been documented in 7% of the elderly population, 14% of CKD patients without dialysis and 42% of adult ESRD patients on hemodialysis (HD) [9]. The prevalence of frailty among the Japanese elderly and CKD population is comparable to the above-mentioned values [12-14]. However, the status of frailty among Japanese HD patients remains unknown.

The aim of this study was to identify the prevalence and predictors of frailty in Japanese HD patients.

## MATERIALS AND METHODS

### Study Design and participants

This study was a multicenter, cross-sectional and observational investigation, which started on October 2015. This study was conducted at 6 institutions with an HD unit, including 5 general hospitals and 1 private clinic: Innoshima General Hospital, Nippon Kokan Fukuyama Hospital, Sumitomo Besshi Hospital, Mihara Shiromachi Hospital, Akaiwa Medical Association Hospital and Sugimoto Clinic. All of the data were obtained by the attending physicians and medical staff at each institution and sent to the Okayama University Graduate School of Medicine, Dentistry and Pharmaceutical Sciences for the analysis. The patient recruitment ended in January 2016.

The subjects were all chronic hemodialysis patients who regularly visited the institutions within the period of this study, and who agreed with the aim and protocol of the present study. The initial exclusion criteria were: (1) refusal to participate, (2) hospitalization due to accidents or sickness, (3) patients who found it difficult to answer the questionnaire due to severe dementia or communication disability and (4) temporary hemodialysis patients or patients who had received hemodialysis for less than 3 months. Finally, we excluded participants who did not completely fill in the frailty contents (which we explain below “*Definition of the frailty phenotype*” paragraph) in the questionnaire completely, from those remaining after the initial exclusion, because the presence or absence of frailty could not be properly assessed.

**Table 1:** Operational definition of the frailty phenotype in the present study

Criteria	Definition
Weight Loss	Unintentional weight loss ≥ 2 kg in the previous year
Poor Endurance	Positive answer to a self-reported question, about how the participant had felt in the last 2 weeks: “Did you feel exhausted without any reason?”
Weakness	Grip strength by gender Males: < 26.0 kg, Females: < 18.0 kg
Slowness	Positive answer to either of two self-reported questions, if participants were asked about their walking speed: “Are you unable to walk at a pace of ≥ 1.0 m/sec?”, “Is it hard for you to cross over a crosswalk within the time allotted?”
Low activity	Negative answer to both of two self-reported questions, on the participants’ activity: “Do you lightly exercise or work at least once a week?”, “Do you regularly play any sports at least once a week?”

Frail: ≥ 3 criteria met. Intermediate or Pre-Frail: 1 or 2 criteria met. Not frail: no criteria met.

### Evaluation measurements and factors

By checking medical records, the following risk factors of each patient were evaluated: body mass index (BMI), hypertension (HTN), dyslipidemia (DLP), DM, smoking habit (SMK), ischemic heart disease (IHD), stroke (STK), peripheral arterial disease (PAD), malignancy (MLG), frequency and quantity of dialysis, data of blood tests, ankle-brachial index (ABI), brachial-ankle plus wave velocity (baPWV), history of bone fracture (BF) and ESRD. The definition of HTN, DLP, DM, IHD, STK and PAD, and measurement of physical domain are described in supplement file. To evaluate the dialysis efficiency, we calculated single-pooled Kt/V (spKt/V) [15]. To evaluate the nutritional status, we calculated normalized protein catabolic rate (nPCR) [16] and Geriatric Nutritional Risk Index (GNRI) [17], and asked participants to fill out the Mini Nutritional Assessment-Short Form (MNA-SF) [18] questionnaire. To evaluate the frailty status, we asked participants to check the appropriate boxes no the frailty phenotype questionnaire which we made according to the “*Definition of the frailty phenotype*” described below.

### Definition of the frailty phenotype

The operational definition of frailty phenotype in the present study was as follows (see details in Table 1): All five criteria were modified from the original Fried CHS frailty phenotype [4], for Japanese population. Weight loss was defined as unintentional weight loss ≥ 2 kg in the previous year, according to an indicator of nutrition for identifying vulnerable older adults in the long-term care insurance system on the Kihon-Checklist, which is a self-reported comprehensive health checklist developed by the Japan Ministry of Health, Labour and Welfare [19]. Poor Endurance was defined by a positive answer to a self-reported question, about how the participant had felt in the last 2 weeks: “Did you feel exhausted without any reason?” which is also taken from the Kihon-Checklist [19]. Weakness was defined using the maximum grip strength by gender according to the Asian Working Group for Sarcopenia criteria (Males: < 26.0 kg, Females: < 18.0 kg) [20]. Slowness was defined by a positive answer to either of two self-reported questions, if participants were asked about their walking speed: “Are you unable to walk at a pace of ≥ 1.0 m/sec?”, “Is it hard for you to cross over a crosswalk within the time allotted?”, according to a previously established cut-off of walking speed <1.0 m/s [14]. Low activity was defined as negative answer to both of two self-reported questions, on the participants’ activity: “Do you lightly exercise or work at least once a week?”, “Do you regularly play any sports at least once a week?”, which was also taken from the same established definition [14]. Individuals who met at least 3 criteria were defined as frailty. Individuals who met 1 or 2 criteria were defined as pre-frailty (intermediate frailty status), and those not meeting any criteria were considered as not-frailty.

### Ethics

This study complied with the Declaration of Helsinki (seventh revision, 2013) on medical protocol and ethics. This was a cross-sectional observational study. Since we collected the data from physicians’ charts and questionnaires filled out by the patients, the Institutional Review Boards at each hospital waived the requirement of written informed consent but requested patients be given the opportunity to refuse enrollment in this study by leaflets or the hospital website. Finally, each ethics committee of the Institutional Review Board approved the protocol (UMIN ID: 000024783).

**Figure 1.**
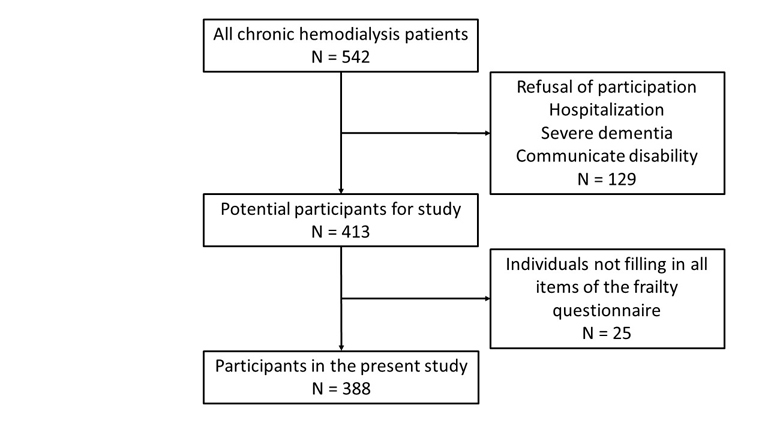
Diagram of participants enrolled in this study.

### Statistical analysis

All data were expressed as the mean ± standard deviation or n (%). Differences among each phenotype were examined by a one-way analysis of variance or chi-squared test. To evaluate the predictors of frailty and pre-frailty, we performed univariate and multivariate logistic regression analyses which estimated the prevalence odds ratio (OR) for frailty relative to not-frailty and for pre-frailty relative to not-frailty. Regarding explanatory variables, we assessed all of the evaluated risk factors for frailty that are described in above “*Evaluation measurements and factors*”. Parameters such as age and the laboratory data were evaluated with both continuous variables and binary variables with known specific cut-off values. For the multivariate logistic regression analysis, we simultaneously introduced independent variables into the several models based on classical risk factors for frailty, such as age, female gender, DM, IHD, STK, PAD, BF, Fall, obesity (BMI ≥ 25.0) or underweight (BMI < 18.5) and MLG. Factors which had strong confounding influence on each other were not included in the same model.

A difference of *P* < 0.05 was taken as statistically significant. All data were analyzed using Sigma Plot for Windows (version 13.0, Systat Software Inc., San Jose, California, USA).

**Figure 2.**
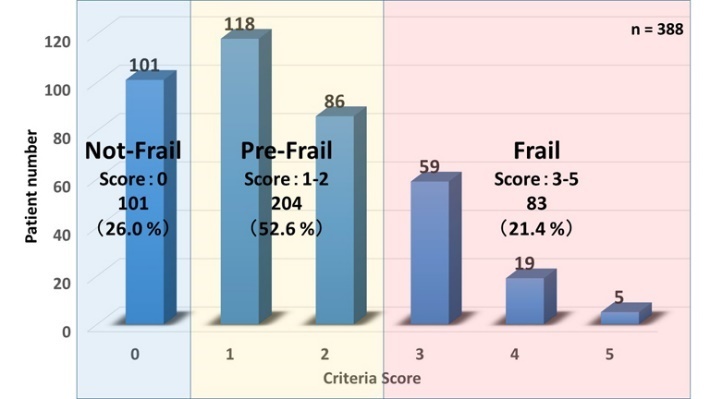
Number of frailty criteria present; Prevalence of Frailty status.

## RESULTS

### Study Participants

As shown in Figure 1, among the 542 patients visiting each institution, 129 did not participate due to refusal and hospitalization due to sickness or difficulty in communication, 25 patients who were unable to properly fill out the frailty questionnaire were excluded. Finally, 388 patients were enrolled in this study. The baseline characteristics of the participants are displayed in Supplementary Table 1. Participants were an average of 67.2 ± 11.9 years old with more male gender (62.4%) than female. Almost all patients received HD for 4 hours in 3 sessions a week. The leading etiology of ESRD was diabetic nephropathy. Those characteristics were similar to the population of typical chronic HD patients in Japan.

### Frailty Prevalence among Dialysis patients

The total frailty criteria score, and prevalence of each frailty phenotype are shown in Figure 2. In total, 21.4% of participants were categorized as frailty, 52.6% as pre-frailty and 26.0% as the subjects without frailty.

**Figure 3.**
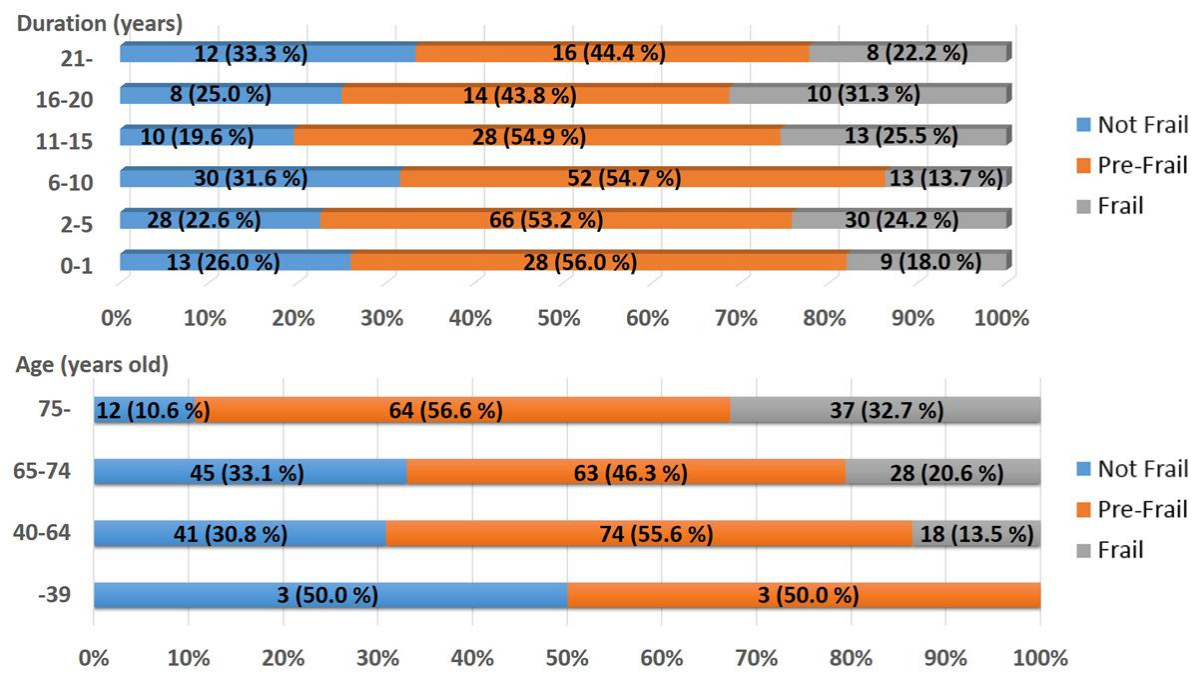
The Ratio of the Frailty Phenotypes according to the Duration of Dialysis and Age. The upper panel shows the ratio of the frailty phenotypes according to the duration of dialysis. The lower panel shows the ratio of the frailty phenotypes according to the age.

**Table 2:** Characteristics of each frailty phenotype.

Variable	Not frail (n = 101)	Pre-Frail (n = 204)	Frail (n = 83)	*P value*
Age, (years)	63.6 ± 11.2	67.5 ± 12.3	71.0 ± 10.2	< 0.001^**^
Gender (male), n (%)	70 (69.3 %)	135 (66.2 %)	37 (44.6 %)	< 0.001^**^
Height, (cm)	162.0 ± 8.9	159.7 ± 9.3	155.2 ± 9.0	< 0.001^**^
Weight, (kg)	56.0 ± 11.1	56.6 ± 12.1	52.9 ± 10.4	0.043^*^
Body mass index, (kg/m^2^)	21.2 ± 3.4	22.1 ± 3.5	21.9 ± 3.8	*0.155*
Brachial circumference, (cm)	25.3 ± 3.1	25.3 ± 3.2	24.8 ± 2.9	*0.481*
Rt. Femoral circumference, (cm)	42.4 ± 5.4	41.5 ± 5.2	40.0 ± 5.0	0.007^**^
Lt. Femoral circumference (cm)	42.5 ± 5.1	41.0 ± 5.1	39.8 ± 4.8	0.006^**^
Grip strength, (kg)	26.5 ± 9.7	22.1 ± 11.1	15.8 ± 7.5	< 0.001^**^
Rt. ABI	1.13 ± 0.19	1.12 ± 0.23	1.07 ± 0.26	*0.123*
Lt. ABI	1.11 ± 0.17	1.10 ± 0.23	1.07 ± 0.24	*0.485*
Hb, (g/dL)	10.8 ± 1.0	10.8 ± 1.0	10.7 ± 1.0	*0.517*
Alb, (g/dL)	3.7 ± 0.8	3.6 ± 0.3	3.5 ± 0.4	0.003^**^
T-Chol, (mg/dL)	163 ± 40	159 ± 41	154 ± 40	*0.363*
UN, (mg/dL)	63.7 ± 14.3	63.2 ± 17.3	60.2 ± 20.1	*0.338*
Cr, (mg/dL)	10.76 ± 5.13	9.44 ± 2.41	8.35 ± 2.17	<0.001^**^
Number of oral medications	9.3 ± 3.2	9.9 ± 3.7	12.1 ± 3.8	< 0.001^**^
Dementia drugs used, n (%)	0 (0.0 %)	5 (2.5 %)	3 (3.6 %)	*0.195*
Smoker (current + former), n (%)	54 (53.5 %)	89 (43.6 %)	30 (36.1 %)	*0.058*
History of falling, n (%)	15 (14.9 %)	52 (25.5 %)	30 (36.1 %)	0.004^**^
Dialysis frequency, (sessions/week)	3.0 ± 0.1	3.0 ± 0.2	3.0 ± 0.3	*0.328*
Dialysis time, (hour/session)	4.1 ± 0.3	4.1 ± 0.3	4.1 ± 0.4	*0.559*
Duration of dialysis, (years)	8.6 ± 7.3	8.3 ± 7.4	9.5 ± 8.4	*0.431*
spKt/V urea	1.48 ± 0.37	1.46 ± 0.39	1.50 ± 0.40	*0.744*
nPCR, (g/kg/day)	0.87 ± 0.12	0.87 ± 0.16	0.84 ± 0.20	*0.336*
GNRI	95.3 ± 14.2	95.6 ± 8.6	93.5 ± 10.1	*0.313*
MNA-SF	11.9 ± 2.1	11.7 ± 1.8	10.2 ± 2.6	<0.001^**^
Etiology of ESRD				
CGN, n (%)	47 (46.5 %)	50 (24.5 %)	20 (24.1 %)	< 0.001^**^
DN, n (%)	24 (23.8 %)	75 (36.8 %)	39 (47.0 %)	0.004^**^
NS, n (%)	9 (8.9 %)	17 (8.3 %)	1 (1.2 %)	*0.066*
PKD, n (%)	2 (2.0 %)	8 (3.9 %)	3 (3.6 %)	*0.667*
Others, n (%)	7 (6.9 %)	22 (10.8 %)	8 (9.6 %)	*0.559*
Unknown, n (%)	12 (11.9 %)	32 (15.7 %)	12 (14.5 %)	*0.673*
HTN, n (%)	76 (75.2 %)	151 (74.0 %)	60 (72.3 %)	*0.901*
DLP, n (%)	16 (15.8 %)	47 (23.0 %)	20 (24.1 %)	*0.281*
DM, n (%)	26 (25.7 %)	89 (43.6 %)	47 (56.6 %)	< 0.001^**^
IHD, n (%)	21 (20.8 %)	48 (23.5 %)	20 (24.1 %)	*0.833*
STK, n (%)	8 (7.9 %)	27 (13.2 %)	19 (22.9 %)	0.013^*^
PAD, n (%)	13 (14.0 %)	29 (16.5 %)	21 (28.4 %)	0.038^*^
MLG, n (%)	7 (6.9 %)	20 (9.8 %)	8 (9.6 %)	*0.695*
BF, n (%)	5 (5.0 %)	22 (10.8 %)	11 (13.3 %)	*0.133*

The data are presented as the mean value ± standard deviation or n (%) of patients. Hb, hemoglobin; Alb, albumin; T-Chol, total cholesterol; UN, urea nitrogen; Cr, creatinine; spKt/V urea, dialysis efficacy; nPCR, normalized protein catabolic rate; GNRI, Geriatric Nutritional Risk Index; MNA-SF, mini nutritional assessment-short form; ESRD, end-stage renal disease; CGN, chronic glomerulonephritis; DN, diabetic nephropathy; NS, nephrosclerosis; PKD, polycystic kidney disease; HTN, hypertension; DLP, dyslipidemia; DM, diabetes mellitus; IHD, ischemic heart disease; STK, stroke; MLG, malignancy; BF, bone fracture. *P* values are obtained by One Way ANOVA test or chi-square test.

* *P* < 0.05,

** *P* < 0.01.

**Table 3:** Univariate predictors of frail and pre-frail.

	Pre-Frail			Frail		
	Odds ratio	95% CI	*P value*	Odds ratio	95% CI	*P value*
Female	1.154	0.691-1.927	0.584	2.807	1.533-5.141	< 0.001^**^
Age, years	1.027	1.007-1.048	0.009^**^	1.072	1.037-1.107	< 0.001^**^
Age ≥ 65 y.o.	1.273	0.784-2.067	0.329	2.788	1.450-5.359	0.002^**^
Age ≥ 75 y.o.	3.390	1.733-6.635	< 0.001^**^	5.966	2.840-12.529	< 0.001^**^
BMI ≥ 25.0	2.121	1.041-4.324	0.038^*^	1.660	0.710-3.883	*0.242*
BMI <18.5	0.654	0.361-1.188	0.163	0.412	0.179-0.949	0.037^*^
DN	1.865	1.088-3.199	0.023^*^	2.844	1.516-5.335	0.001^**^
HTN	0.974	0.561-1.692	0.925	0.858	0.444-1.660	*0.649*
DLP	1.632	0.872-3.053	0.125	1.687	0.810-3.513	*0.163*
DM	2.272	1.343-3.842	0.002^**^	3.776	2.021-7.018	< 0.001^**^
IHD	1.195	0.669-2.134	0.547	1.209	0.603-2.425	*0.592*
STK	1.783	0.779-4.082	0.171	3.451	1.424-8.364	0.006^**^
PAD	1.214	0.598-2.466	0.592	2.438	1.125-5.287	0.024^*^
MLG	1.468	0.599-3.595	0.401	1.432	0.497-4.129	*0.506*
BF	2.334	0.857-6.357	0.097	2.933	0.976-8.816	*0.055*
Fall	1.991	1.057-3.751	0.033^*^	3.208	1.580-6.514	0.001^**^
Smoking	0.680	0.419-1.105	0.119	0.472	0.260-0.857	0.014^*^
NOM	1.044	0.974-1.119	0.221	1.256	1.140-1.383	< 0.001^**^
Hypo-Alb	1.305	0.754-2.261	0.341	1.913	1.010-3.622	0.047^*^
Hypo-Chol	1.086	0.671-1.758	0.736	1.382	0.771-2.477	*0.278*
spKt/V ≥1.80	0.844	0.464-1.535	0.578	1.290	0.647-2.572	*0.469*
spKt/V <0.80	3.553	0.431-29.281	0.239	1.220	0.075-19.798	*0.889*
nPCR <0.90	1.018	0.629-1.646	0.944	1.230	0.681-2.223	*0.492*
GNRI ≤91	0.901	0.542-1.497	0.687	1.509	0.829-2.747	*0.178*
MNA-SF ≤11	1.316	0.804-2.154	0.275	3.958	2.135-7.338	< 0.001^**^

CI, confidence interval; BMI, body mass index; DN, diabetic nephropathy; HTN, hypertension; DLP, dyslipidemia; DM, diabetes mellitus; IHD, ischemic heart disease; STK, stroke; PAD, peripheral arterial disease; MLG, malignancy; BF, bone fracture; NOM, number of oral medicine; Hypo-Alb, hypoalbuminemia; Hypo-Chol, hypocholesterolemia; spKt/V, dialysis efficacy; nPCR, normalized protein catabolic rate; GNRI, geriatric nutritional risk index; MNA-SF, mini nutritional assessment-short form. The odds ratio and P values were obtained by a univariate logistic regression analysis.

* P < 0.05,

** P < 0.01.

In Supplementary Figure 1, the population and prevalence of each frailty criterion are shown. In this study, the prevalence of each criterion was all significantly higher in the frailty group than that in the pre-frailty group. Among the subjects with frailty, the prevalence of “Poor Endurance”, “Slowness” and “Low Activity” were higher than that of “Weakness” and “Weight Loss”. In contrast, among the subjects with pre-frailty, “Low Activity” had the highest prevalence.

There was no relationship between the duration of HD and frailty status as shown in Figure 3. On the other hand, the number of subjects with frailty increased steadily with age (Figure 3 and Supplementary Figure 2). Among the elderly subjects, the population ≥ 75 years old had the highest prevalence of frailty (Figure 3). Furthermore, 13.5% of non-elderly patients were frailty, despite the fact that frailty is a geriatric syndrome.

In Table 2, the characteristics of each frailty phenotype are shown. The average age of subjects increased significantly as the frailty stages progressed, and frailty was more prevalent in females than in males. The subjects with frailty or pre-frailty had lower body weight and lower grip strength than those without frailty. Serum albumin concentrations were slightly lower in the subjects with frailty and pre-frailty than in those without frailty. The subjects with frailty were under the treatment of polypharmacy: the mean number of tablets administered to the patients with frailty, pre-frailty and those without frailty were 12.1, 9.9 and 9.3 respectively. There was no marked difference in the frequency or efficiency of hemodialysis among each phenotype.

### Nutritional Status

**Figure 4.**
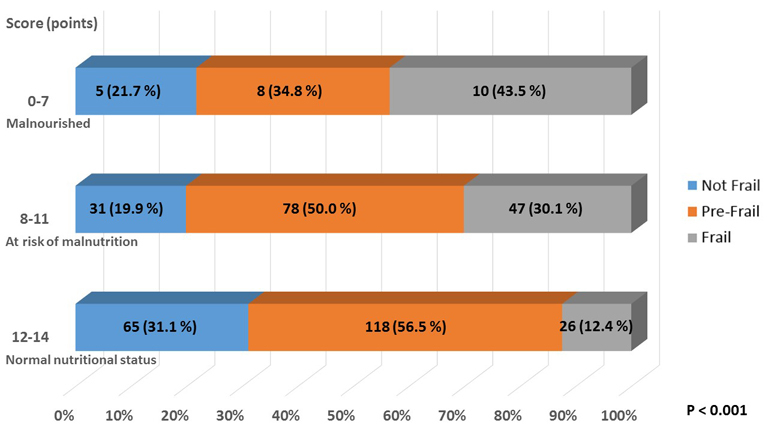
The Ratio of Frailty Phenotypes according to the MNA-SF Score. P value was obtained by chi-square test.

**Figure 5.**
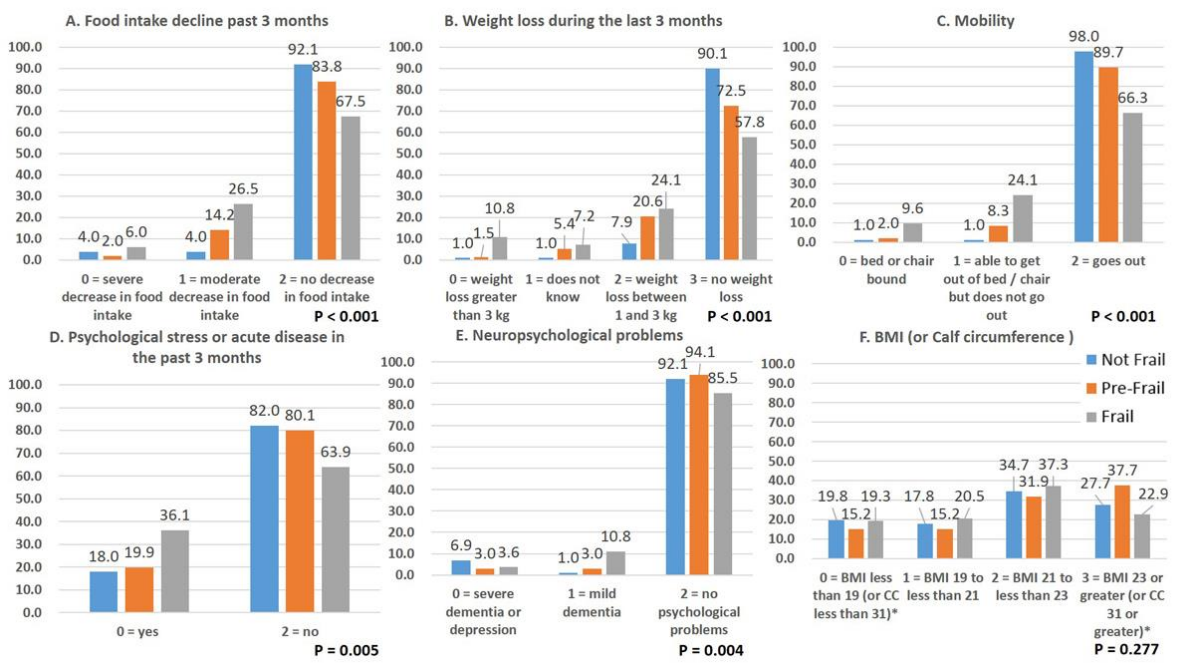
Details of MNA-SF Score. Panel (A) shows the proportion of MNA-SF criteria A: “Has food intake declined over the past 3 months due to loss of appetite, digestive problems, chewing or swallowing difficulties?” in each frailty status. Panel (B) shows the proportion of MNA-SF criteria B: Weight loss during the last 3 months, in each frailty status. Panel (C) shows the proportion of MNA-SF criteria C: Mobility, in each frailty status. Panel (D) shows the proportion of MNA-SF criteria D: “Has suffered psychological stress or acute disease in the past 3 months?” in each frailty status. Panel (E) shows the proportion of MNA-SF criteria E: Neuropsychological problems, in each frailty status. (F) shows the proportion of MNA-SF criteria F1: Body Mass Index or F2: Calf circumference, in each frailty status. P values were obtained by chi-square tests.

There was unexpectedly no marked difference in nPCR or GNRI according to the frailty status. Only MNA-SF differed significantly between subjects with frailty and others. The prevalence of patients according to MNA-SF scores are shown in Figure 4. The subjects with frailty increased as malnutrition developed. The details of the factors which comprise MNA-SF are shown in Figure 5(A)-(F). The scores tended to be lower for the subjects with frailty or pre-frailty in each category except for BMI.

### Accumulation of the Risk Factors for Frailty

**Figure 6.**
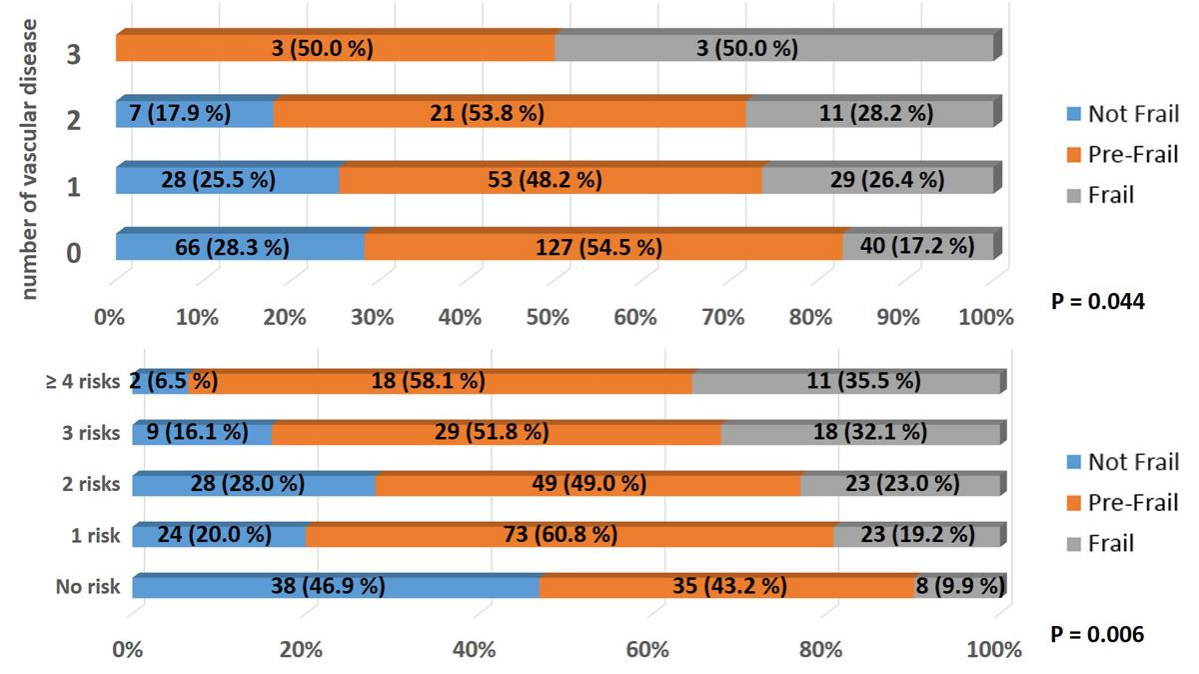
Prevalence of Frailty Phenotypes according to the Number of Cardiovascular Disease and the Number of General Risk Factors. The upper panel shows the prevalence of frailty phenotypes according to the number of the cardiovascular disease. The lower panel shows the prevalence of frailty phenotypes according to the number of the general risk factors for frailty. Cardiovascular diseases are ischemic heart disease, stroke and peripheral artery disease. The general risk factors for frailty are ischemic heart disease, stroke, peripheral artery disease, malignancy, obesity, bone fracture, hypoalbuminemia and/or diabetes. P value were obtained by chi-square tests.

When comparing the number of risk factors for frailty, patients with frailty appeared to have more risk factors than others. Figure 6 shows the prevalence of each frailty status, according to the number of cardiovascular diseases (IHD, STK and / or PAD). The proportion of frailty steadily increased as the number of cardiovascular disease increased, with borderline significance. In addition, the prevalence of frailty according to the number of general risk factors for frailty (cardiovascular diseases, MLG, obesity, BF, hypoalbuminemia and/or DM), is also shown in Figure 6. The proportion of patients with frailty significantly increased as the number of risk factors increased. These results imply that the accumulation of risk factors easily leads patients to frailty.

**Table 4:** Multivariate Analysis of predictors for frail and pre-frail.

	Pre-Frail			Frail		
	Odds ratio	95% CI	*P value*	Odds ratio	95% CI	*P value*
Female	1.380	0.759-2.509	*0.292*	3.661	1.398-9.588	0.008^**^
Age	1.026	1.004-1.049	0.019^*^	1.065	1.014-1.119	0.013^*^
BMI ≥ 25.0	2.463	1.079-5.623	0.032^*^			
BMI <18.5	0.110	0.0293-0.416	0.001^**^
NOM	1.038	0.954-1.130	*0.383*	1.351	1.163-1.570	< 0.001^**^
DM	2.274	1.203-4.296	0.011^*^	2.765	1.081-7.071	0.034^*^
IHD	0.882	0.414-1.877	*0.744*	1.026	0.331-3.181	*0.964*
STK	1.383	0.524-3.653	*0.513*	3.136	0.824-11.929	*0.094*
PAD	0.775	0.337-1.783	*0.548*	2.314	0.730-7.332	*0.154*
MLG	1.382	0.517-3.692	*0.519*	0.877	0.170-4.535	*0.876*
BF	1.612	0.469-5.541	*0.449*	1.415	0.247-8.097	*0.696*
Fall	1.176	0.559-2.473	*0.670*	1.526	0.468-4.978	*0.483*
MNA-SF ≤11	1.448	0.817-2.567	*0.205*	7.405	2.732-20.072	< 0.001^**^

CI, confidence interval; BMI, body mass index; NOM, number of oral medicine; DM, diabetes mellitus; IHD, ischemic heart disease; STK, stroke; PAD, peripheral arterial disease; MLG, malignancy; BF, bone fracture; Hypo-Alb, hypoalbuminemia; MNA-SF, mini nutritional assessment-short form. The above data were adjusted for all items written in the column. The odds ratio and P values were obtained by a multivariate logistic regression analysis.

* P < 0.05,

** P < 0.01.

**Table 5:** Multivariate Analysis of predictors for frail and pre-frail, categorized as elderly criteria.

	Pre-Frail			Frail Model 1			Frail Model 2		
	Odds ratio	95% CI	*P value*	Odds ratio	95% CI	*P value*	Odds ratio	95% CI	*P value*
Female	1.650	0.889-3.064	*0.113*	3.581	1.395-9.190	0.008^**^	3.733	1.413-9.858	0.008^**^
Age ≥ 65 y.o.				2.429	0.894-6.599	*0.082*			
Age ≥ 75 y.o.	3.928	1.827-8.447	<0.001^**^				4.892	1.715-13.955	0.003^**^
BMI ≥ 25.0	2.731	1.196-6.328	0.017^*^						
BMI <18.5				0.104	0.0281-0.383	< 0.001^**^	0.129	0.0335-0.499	0.003^**^
NOM	1.035	0.949-1.130	*0.433*	1.344	1.161-1.556	< 0.001^**^	1.389	1.192-1.618	<0.001^**^
DM	2.704	1.400-5.226	0.003^**^	2.864	1.125-7.294	0.027^*^	2.811	1.069-7.387	0.036^*^
IHD	0.886	0.410-1.916	*0.758*	1.076	0.355-3.259	*0.897*	0.929	0.293-2.950	*0.901*
STK	1.260	0.467-3.403	*0.648*	3.392	0.921-12.493	*0.066*	3.414	0.921-12.658	*0.066*
PAD	0.687	0.292-1.619	*0.391*	2.176	0.681-6.946	*0.189*	2.613	0.805-8.481	*0.110*
MLG	1.450	0.528-3.979	*0.471*	0.916	0.175-4.788	*0.917*	0.910	0.174-4.763	*0.911*
BF	1.662	0.464-5.948	*0.435*	1.660	0.297-9.296	*0.564*	1.368	0.226-8.296	*0.733*
Fall	1.180	0.551-2.529	*0.670*	1.598	0.483-5.285	*0.443*	1.653	0.505-5.409	*0.406*
Hypo-Alb	1.202	0.618-2.339	*0.588*	1.884	0.684-5.189	*0.221*	1.732	0.617-4.858	*0.297*
MNA-SF ≤11	1.529	0.884-2.866	*0.121*	7.207	2.702-19.221	< 0.001^**^	7.609	2.742-21.115	<0.001^**^

CI, confidence interval; y.o., years old; BMI, body mass index; NOM, number of oral medicine; DM, diabetes mellitus; IHD, ischemic heart disease; STK, stroke; PAD, peripheral arterial disease; MLG, malignancy; BF, bone fracture; Hypo-Alb, hypoalbuminemia; MNA-SF, mini nutritional assessment-short form. The above three models were adjusted for all items written in the column. The odds ratio and P values were obtained by a multivariate logistic regression analysis.

* P < 0.05,

** P < 0.01

### Predictors of Frailty

The predictors of frailty and pre-frailty evaluated by univariate and multivariate logistic regression analysis, are shown in Table 3, 4 and 5. The factors independently associated with frailty were the following: female gender (OR = 3.661, 95% CI 1.398-9.588), age (OR = 1.065, 95% CI 1.014-1.119), age ≥ 75 years old (OR = 4.892, 95% CI 1.715-13.955), BMI < 18.5 (OR = 0.110, 95% CI 0.0293-0.416), number of medications being taken (OR = 1.351, 95% CI 1.163-1.570), DM (OR = 2.765, 95% CI 1.081-7.071) and MNA-SF ≤ 11 (OR = 7.405, 95% CI 2.732-20.072). There were no significant relationships among obesity (BMI ≥ 25.0), efficiency of hemodialysis, nPCR and GNRI, in any combination of multivariate regression model (data not shown).

### Association between physical domain and Frailty

The physical relationships with frailty are shown Supplementary Table 2 and 3. The physical domains significantly associated with frailty were height, weight, femoral circumference and grip strength. However, after adjustment for age and sex, only grip strength retained a significant independent association with frailty. In addition, there was a significant negative correlation between grip strength and the frailty phenotype score. These findings suggest that bodily functions might contribute to frailty in respects other than the physical constitution or muscle mass.

## DISCUSSION

To our knowledge, this is the first investigation concerning the prevalence of frailty among Japanese prevalent patients with chronic HD. This study demonstrated that the proportion of patients meeting the self-reported functional-based definition of frailty, was markedly higher than that of the community-dwelling elderly population [13, 14]: approximately 3 to 4 folds higher. In contrast, the prevalence of frailty with HD patients in this study was relatively lower than that noted in previous studies in other developed countries: 21.4% in this study vs around 42-73% in other developed countries [9, 21-25], despite that Japanese dialysis population are older compared with other developed countries [26]. In addition, the proportion of HD patients with frailty was higher among non-elderly individuals than among those in the community-dwelling population. Predictors of frailty were almost the same as previously reported [3, 21, 27]. Age (especially 75 years old and above), female gender, the number of medicine, DM and MNA-SF ≤ 11 (at risk of malnutrition) were independently and significantly associated with frailty. Furthermore, the accumulation of risk factors for frailty was observed in frail patients. These risk factors are generally related to disability, mortality and several complications. Therefore, this indicates that the patients with frailty might be associated with an increased prevalence of adverse health outcomes in patients with HD. Although we have not yet performed a further longitudinal investigation, these findings suggest that frailty might affect the prognosis and quality of life (QOL) of patients with HD.

### CKD and Dialysis

CKD patients easily develop “Protein Energy Wasting (PEW)”, accompanied by malnutrition and a reduction in the muscle mass [28]. Loss of muscle mass, as investigated by the biological impedance method, was frequently observed with a reduced eGFR or albuminuria-positive patients [29]. Dialysis patients might age 15 years faster than healthy people, observed in the Gompertz equation model [30]. Several reports are available concerning that toxic factors in uremic state accelerated aging and led to a progressively impaired organ function [31]. In addition, anorexia caused by uremic toxins, dialysate and urine nutrient losses, catabolic effect, chronic low-grade inflammation, deficiency or resistance to anabolic hormone and physical inactivity have been reported to induce PEW and frailty [11]. Furthermore, the physical activity is known to decrease in HD patients, due to the maintenance of HD and fatigue after HD, leading to physical deconditioning.

Accordingly, these findings indicate that CKD and ESRD patients are particularly susceptible to frailty. Indeed, the prevalence of frailty increased as CKD stage progressed [32]. In analyses using data from the US Renal Data System (USRDS), 67.7% of the 2275 dialysis patients were considered as frailty [21]. Furthermore, in the population undergoing HD, there were a substantial number of patients with frailty in the non-elderly group, and frailty is a strong and independent predictor of mortality and hospitalizations, regardless of age [21, 24]. In our findings, muscular strength and muscle mass decreased and the accumulation of risk factors for frailty including cardiovascular disease was observed in patients with frailty. These factors are generally related to disability, mortality and other complications. Thus, it is important to detect frailty and intervene at an early stage, before it progresses to disability and leads to adverse health outcomes.

### Comparison with other countries

The prevalence of frailty in the present study was almost half of that noted in other developed countries. Two studies using the CHS frailty phenotype model, in which the definition of frailty is almost the same as our own, estimated the prevalence of frailty in the community-dwelling elderly population in Japan to be 6.9% and 9.3%, respectively [13, 14]. When restricted to the reports using the CHS frailty phenotype model, the average prevalence of frailty was 9.9% (95% CI: 9.6-10.2%) [33]. These results suggest that the characteristic background of the general population may not differ markedly between Japanese and Western population.

The increased longevity of Japanese HD patients in comparison to HD patients in other developed countries may be one of the reasons for the discrepancy in the prevalence of frailty in the present study and other studies on frailty in patients HD [34]. Recently, it was reported that the health-related quality life (HR-QOL) was associated with mortality in HD patients, and DOPPS investigators found a higher physical component score of HR-QOL among Japanese patients with HD than that among participants from other countries [35]. Indeed, it was reported that the status of frailty was closely associated with HR-QOL [36]. These factors may underlie the differences in the prevalence of frailty by country.

The background characteristics of the participants in our study (including age, proportion of gender, etiology of ESRD, frequency and efficiency of hemodialysis) were almost the same as those reported by The Japanese Society for Dialysis Therapy at the end of 2013. Although we have not examined this point yet, the HR-QOL of the patients in the present study may be similar to reported Japanese population.

### Nutrition assessment

The nPCR is valid as a measure of protein intake in HD patients with a neutral nitrogen balance. Several studies have suggested that a poor nPCR is associated with mortality [37, 38]. The GNRI has also been reported to be a significant predictor for mortality in HD patients [17]. However, in the present study, no significant differences in the scores of these two measurements were confirmed among each of frailty phenotype groups. Furthermore, there were only slightly differences in albumin and urea nitrogen levels, which used in the calculation formula as shown in Table 2. Indeed, in Japan, chronic HD patients regularly receive nutritional counselling according to the guidelines of the national society of dialysis and most of the patients eat the lunch and / or dinner provided by their dialysis facilities when they visit to receive HD. Thus, the difference in nutrition among the patients might be slight. This may be one of the reasons underlying the discrepancy in the prevalence of frailty between the present study and studies from other countries.

In this study, the MNA-SF was the only significantly influential tool. In a multivariate analysis, MNA-SF ≤ 11, a cut-off point of “at risk of malnutrition”, also had a significantly higher OR than other factors. In the several reports validating the MNA-SF for use in elderly subjects with frailty, the MNA-SF appeared to be an effective tool for both malnutrition and frailty screening. One report showed that the 11 points cut-off, which is commonly considered to indicate a risk of malnutrition, provided the best correct classification ratio (91.4%), with a sensitivity = 94.0% and specificity = 83.3% [18]. Other reports also showed a close association between the MNA-SF and frailty [39-41]. Thus, in line with previous reports, we confirmed that MNA-SF is an influential tool in our research. MNA-SF consists of 6 categories ( “declined food intake due to loss of appetite, digestive problem, chewing or swallowing difficulties”, “reduced weight during the last 3 months”, “Mobility”, “psychological stress or acute disease in the past 3 months”, “neuropsychological problems” and “BMI”). In the clinical setting, the MNA-SF proved particularly effective because we are able to evaluate the risk of malnutrition repeatedly in a short period. In addition, if the score of a component of MNA-SF is low, proper intervention should be provided in accordance with the category. For example, if a patient has a low score for food intake, nutritional support, dental intervention or dysphagia rehabilitation should be provided. Depending on the low score category, mental and physical interventions may be indicated in other cases. Thus, MNA-SF is beneficial, both as a screening tool for frailty, and as an assessment tool for intervention.

### Limitations

The present study is associated with several limitations. First, this was a cross-sectional study. Therefore, the risk for hospitalization or death in the population with frailty was not clarified in this study. Second, depression and cognitive decline were not assessed, therefore the influences of these factors on the findings in the current study could not be determined; however, the concept of frailty itself includes aspects of depression and cognitive decline in part. Third, we did not conduct a detailed evaluation of the physical function, such as walking speed and chair standing up time. Further, we did not check the exercise tolerability. Fourth, we did not evaluate the subjects’ physical body composition, such as their muscle mass, body fat or edema, using a bioelectrical impedance analysis or dual energy X-ray absorptiometry; however, the brachial or femoral circumferences were measured, which we feel can be substituted as measures of the body composition. Fifth, the results of a subjective evaluation were the primary outcome and frailty was not evaluated objectively. Accordingly, the actual incidence of frailty might have been underestimated.

### Conclusion

In summary, we confirmed the prevalence and predictors of frailty in a Japanese population with HD. Although the prevalence of frailty in the present study was lower than that noted in previous studies in Western developed countries, the prevalence was still extremely high in comparison to the general population regardless age, and frailty was associated with the accumulation of risk factors. The early detection and intervention are likely more important for preventing the adverse outcomes and a poor QOL in patients with HD accordingly. Since patients with HD regularly visit the institution 3 times a week, nutritional and physical intervention are easy to deliver, and they can undergo repeated evaluations of their frailty status. Further detailed assessments, including a prospective longitudinal study and interventional assessment will be required to improve the prognosis and QOL of patients with ESRD.

## Supplementary materials

Supplementary Figure 1 Frequency of individual criterion.

Supplementary Figure 2 The Ratio of Frailty Phenotypes for Each Ages, displayed per 10 years.

**Supplementary Table 1:** The baseline characteristics of all participants.

Variable	Completely filled (n = 388)	Incompletely filled (n = 25)	P-Value
Age, (years)	67.2 ± 11.9	71.9 ± 11.2	0.054
Gender (male), n (%)	242 (62.4 %)	14 (56.0 %)	0.672
Height, (cm)	159.4 ± 9.4	158.9 ± 9.4	0.817
Weight, (kg)	55.7 ± 11.5	54.3 ± 11.3	0.561
Body mass index, (kg/m ^2^)	21.8 ± 3.5	21.3 ± 3.4	0.483
Hb, (g/dL)	10.8 ± 1.0	10.9 ± 1.0	0.587
Alb, (g/dL)	3.6 ± 0.5	3.6 ± 0.3	0.845
T-Chol, (mg/dL)	159 ± 41	159 ± 41	0.979
UN, (mg/dL)	62.7 ± 17.2	68.2 ± 16.1	0.117
Cr, (mg/dL)	9.55 ± 3.39	8.48 ± 1.81	0.119
Number of medications	10.2 ± 3.7	10.2 ± 4.1	0.941
Dialysis frequency, (sessions/week)	3.0 ± 0.2	3.0 ± 0.0	0.944
Dialysis time, (hours/session)	4.1 ± 0.4	4.1 ± 0.6	0.655
Duration of dialysis, (years)	8.7 ± 7.6	5.5 ± 6.0	0.036
spKt/V urea	1.29 ± 0.34	1.30 ± 0.43	0.487
nPCR, (g/kg/day)	0.87 ± 0.16	0.89 ± 0.17	0.417
GNRI	95.2 ± 10.3	94.5 ± 8.2	0.766
MNA-SF	11.5 ± 2.2	10.4 ± 2.6	0.017
Brachial circumference, (cm)	25.2 ± 3.1	25.3 ± 3.9	0.918
Rt. Femoral circumference, (cm)	41.4 ± 5.3	42.0 ± 5.4	0.624
Lt. Femoral circumference (cm)	41.1 ± 5.1	42.3 ± 4.9	0.314
Grip strength, (kg)	22.0 ± 10.7	23.6 ± 9.4	0.501
Blank responses in questionnaire			
0	388 (100 %)	0 (0 %)	
1	0 (0 %)	12 (85.7 %)	
2	0 (0 %)	0 (0 %)	
3	0 (0 %)	2 (14.3 %)	
“Yes” to frailty questionnaire			
0	101 (26.0 %)	9 (64.3 %)	
1	118 (30.4 %)	8 (57.1 %)	
2	86 (22.2 %)	6 (42.9 %)	
3	59 (15.2 %)	2 (14.3 %)	
4	19 (4.9 %)	0 (0 %)	
5	5 (1.3 %)	0 (0 %)	

The data are presented as the mean ± standard deviation or n (%) of patients. Hb, hemoglobin; Alb, albumin; T-Chol, total cholesterol; UN, urea nitrogen; Cr, creatinine; eKt/V, dialysis efficacy; nPCR, normalized protein catabolic rate; ESRD, end-stage renal disease; CGN, chronic glomerulonephritis; DN, diabetic nephropathy; NS, nephrosclerosis; PKD, polycystic kidney disease. Completely filled group are the participants enrolled in this study. P values were determined using the chi-squared test.

**Supplementary Table 2:** Physical Association of Frail and Pre-Frail.

	Pre-Frail			Frail		
	Odds ratio	95% CI	*P value*	Odds ratio	95% CI	*P value*
Univariate Analysis
Height, cm	1.008	0.987-1.030	0.438	0.938	0.912-0.965	<0.001 ^**^
Weight, kg	1.016	0.998-1.034	0.084	0.972	0.949-0.994	0.015 ^*^
BMI, kg/m ^2^	1.042	0.984-1.103	0.158	1.011	0.945-1.082	*0.784*
BC, cm	1.028	0.964-1.097	0.397	0.954	0.880-1.033	*0.245*
Mean FC, cm	1.007	0.970-1.045	0.708	0.933	0.888-0.979	0.005 ^**^
Larger FC, cm	1.004	0.966-1.043	0.851	0.931	0.886-0.978	0.004 ^**^
Smaller FC, cm	1.007	0.968-1.048	0.727	0.927	0.881-0.976	0.004 ^**^
Grip Strength, kg	0.963	0.941-0.986	0.002 ^**^	0.865	0.825-0.907	<0.001 ^**^
Multivariate Analysis
Grip Strength, kg ^†^	0.964	0.935-0.993	0.016 ^*^	0.859	0.810-0.911	<0.001 ^**^

CI, confidence interval; BMI, body mass index; BC, brachial circumference; FC, femoral circumference BC is the brachial circumference of dominant arm. The mean FC is the mean circumference of both femurs. A larger FC indicates a larger circumference for both femurs. A smaller FC indicates a smaller circumference for both femurs. The grip strength represents the grip strength of the dominant arm. The odds ratio and P values were obtained by a univariate logistic regression analysis.

* P < 0.05,

** P < 0.01.

† adjusted for age and sex.

**Supplementary Table 3:** Correlation between the frailty phenotype score and physical domain.

	Correlation Coefficient: r	*P value*
Height, cm	-0.238	<0.001 ^**^
Weight, kg	-0.119	0.02 ^*^
BMI, kg/m ^2^	0.0257	0.614
Grip Strength, kg	-0.340	<0.001 ^**^
BC, cm	-0.0780	0.127
Mean FC, cm	-0.165	<0.001 ^**^
Larger FC, cm	-0.193	<0.001 ^**^
Smaller FC, cm	-0.182	<0.001 ^**^

BMI, body mass index; BC, brachial circumference; FC, femoral circumference. BC is the brachial circumference of dominant arm. The mean FC is the mean circumference of both femurs. A larger FC indicates a larger circumference for both femurs. A smaller FC indicates a smaller circumference for both femurs. The grip strength represents the grip strength of the dominant arm. Correlation coefficient: the r and P values were obtained by Pearson product moment correlation.

* P < 0.05,

** P < 0.01.
