# Supplementary figures and images for: The Prevalence of Frailty and its Associated Factors in Japanese Hemodialysis Patients
Source: Aging Dis. 2018 Apr 1;9(2):192–207. doi: 10.14336/AD.2017.0429 (PMC5963342; doi:10.14336/AD.2017.0429)

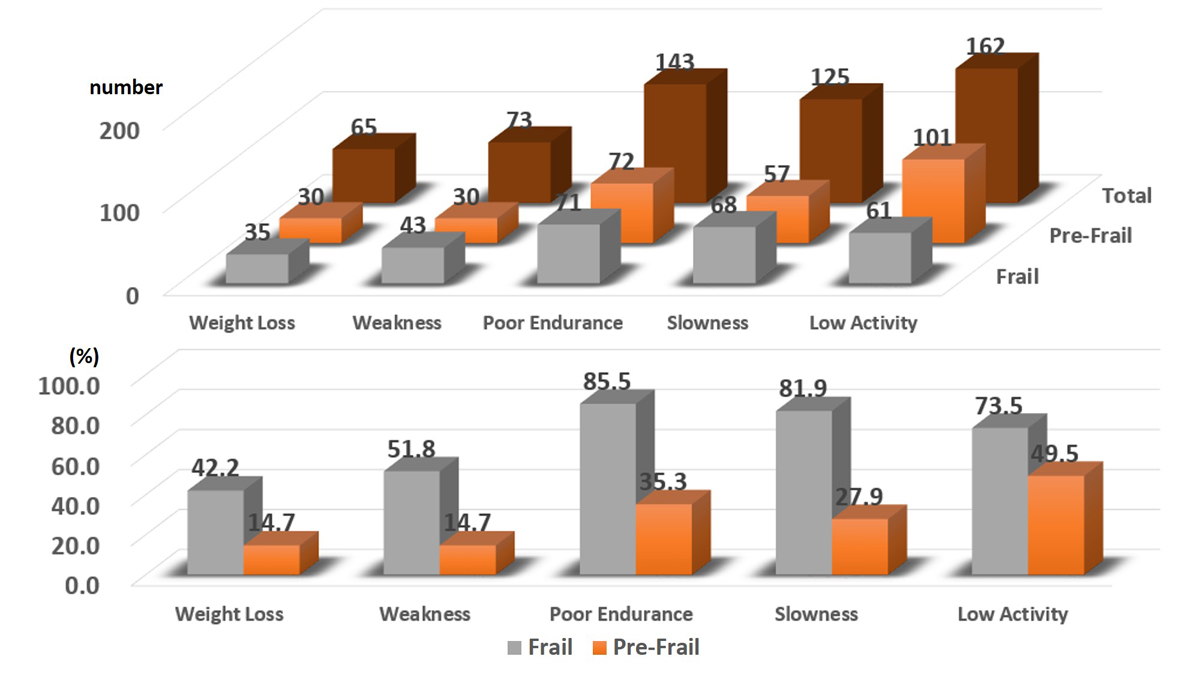

Supplement: Supplementary Figure 1 — Frequency of individual criterion. [file ad-9-2-192-g7.jpg]

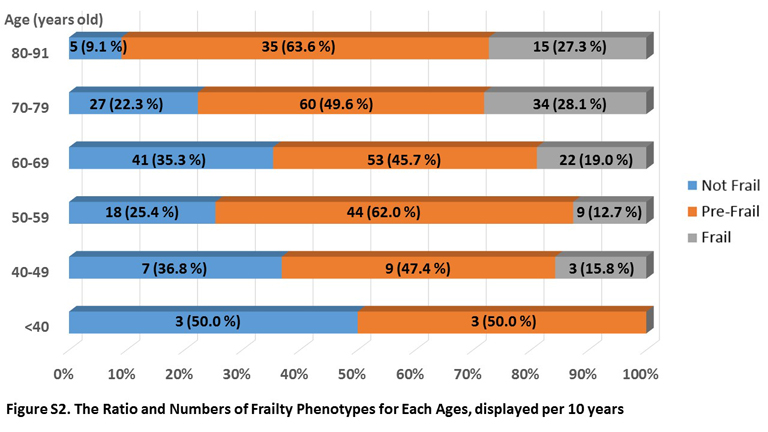

Supplement: Supplementary Figure 2 — The Ratio of Frailty Phenotypes for Each Ages, displayed per 10 years. [file ad-9-2-192-g8.jpg]
